# Comparing Screening Instruments to Predict Posttraumatic Stress Disorder

**DOI:** 10.1371/journal.pone.0097183

**Published:** 2014-05-09

**Authors:** Joanne Mouthaan, Marit Sijbrandij, Johannes B. Reitsma, Berthold P. R. Gersons, Miranda Olff

**Affiliations:** 1 Department of Psychiatry, Centre for Anxiety Disorders, Research Group Psychotrauma, Academic Medical Centre, Amsterdam, The Netherlands; 2 Clinical Psychology, VU University, Amsterdam, The Netherlands; 3 EMGO Institute for Health and Care Research, Amsterdam, The Netherlands; 4 Department of Clinical Epidemiology, Biostatistics and Bioinformatics, Academic Medical Centre, Amsterdam, The Netherlands; 5 Julius Centre for Health Sciences and Primary Care, University Medical Centre, Utrecht, The Netherlands; 6 Arq Psychotrauma Expert Group, Diemen, The Netherlands; University of Stellenbosch, South Africa

## Abstract

**Background:**

Following traumatic exposure, a proportion of trauma victims develops posttraumatic stress disorder (PTSD). Early PTSD risk screening requires sensitive instruments to identify everyone at risk for developing PTSD in need of diagnostic follow-up.

**Aims:**

This study compares the accuracy of the 4-item SPAN, 10-item Trauma Screening Questionnaire (TSQ) and 22-item Impact of Event Scale-Revised (IES-R) in predicting chronic PTSD at a minimum sensitivity of 80%.

**Method:**

Injury patients admitted to a level-I trauma centre (*N* = 311) completed the instruments at a median of 23 days and were clinically assessed for PTSD at 6 months. Areas under the curve and specificities at 80% sensitivity were compared between instruments.

**Results:**

Areas under the curve in all instruments were adequate (SPAN: 0.83; TSQ: 0.82; IES-R: 0.83) with no significant differences. At 80% sensitivity, specificities were 64% for SPAN, 59% for TSQ and 72% for IES-R.

**Conclusion:**

The SPAN, TSQ and IES-R show similar accuracy in early detection of individuals at risk for PTSD, despite differences in number of items. The modest specificities and low positive predictive values found for all instruments could lead to relatively many false positive cases, when applied in clinical practice.

## Introduction

A proportion of individuals exposed to a traumatic event develops psychopathology, such as posttraumatic stress disorder (PTSD) [1]. PTSD consists of symptoms of intrusion, avoidance, and hyperarousal. It is a disabling disorder which may lead to chronic psychiatric morbidity and loss of normal daily functioning [2], [3]. Traumatic injury is one of the most common traumatic events worldwide, accounting for 9% of global mortality [4]. Reported rates of PTSD within the first 6 months of injury range between 10–20% [1], [2], [5]. Early interventions for everyone involved in the traumatic event are unsuccessful in preventing PTSD (e.g., psychological debriefing) [6], whereas there is evidence that early treatment of acute PTSD with trauma-focused cognitive behavioural therapy can ward off a chronic course of PTSD [7], [8]. To identify trauma survivors at risk for PTSD in need of early treatment, the use of self-report instruments has been proposed [9], such as the SPAN [10], the Trauma Screening Questionnaire (TSQ) [11] and the Impact of Event Scale-Revised (IES-R) [12]. These instruments may be used as selection tools as part of a “triage” [13] or “screen and treat” [14] strategy. Only those individuals scoring above the cut-off are referred for further diagnostics. If the instrument is sufficiently accurate, such a strategy may save resources and costs [13].

In evaluating the accuracy of early screening instruments, its sensitivity (i.e., the probability that someone with PTSD has a positive test result) and specificity (i.e., the probability that someone without PTSD has a negative result) are important. The higher the sensitivity, the lower the specificity, and vice versa. In studies of diagnostic accuracy, an optimal cut-off point is usually chosen based on the trade-off between sensitivity and specificity [15]. However, the post-hoc nature in choosing an optimal cut-off has resulted in many different cut-offs reported across studies and populations. This limits comparability between screening instruments, and may complicate the professionals' choice for an early PTSD risk screening instrument. Related to sensitivity and specificity, but more important for clinical practice, are the positive predictive value (PPV) and the negative predictive value (NPV) of a screening instrument. The PPV refers to the proportion of people scoring positive on the screening instrument who indeed have or will develop PTSD, whereas the NPV refers to the proportion of people scoring negative on the screener who do not have or will not develop PTSD. PPV and NPV are both dependent on the population prevalence of a disorder. Typically, when the prevalence of a disorder in the population is low, as is the case in PTSD after injury (10-20%, [1], [2], [5]), PPV will be low and NPV will be high.

Which levels of sensitivity and specificity are acceptable, depends on the aim of administering the screening instrument and the costs and benefits of the decision based on the screening instrument [15]. Thus, there are no standard sensitivity and specificity levels that are considered acceptable. However, with respect to early PTSD risk screening after injury, a few recommendations can be made. First, in the early phase following traumatic injury, sensitivity may be important, to identify as many cases as possible that may be referred for more extensive diagnostic examination. In addition, it has been argued that in populations with a low condition prevalence, more sensitive screening instruments may be especially useful in selecting individuals for further assessment because PPV in that population will be low whereas NPV will be high [16]. Since previous studies in injury victims have shown that sensitivities of early PTSD risk screening instruments vary between 80% to 90% (for a review, see O'Donnell et al. [17]), it may be argued that for being acceptable as an early PTSD risk screener, an instrument should at least have a sensitivity of 80%. To illustrate, when 10% of a trauma population of 1,000 individuals will develop PTSD, 80% sensitivity would imply that 80 PTSD cases will be correctly detected and 20 PTSD cases remain unnoticed by the test. An important question, then, is which early PTSD risk screening instrument performs best at this sensitivity level?

Until now, most of the early PTSD risk screening instruments were developed to predict acute PTSD, but were not evaluated on their accuracy in predicting a chronic PTSD diagnosis. Yet, from a clinical and cost-effectiveness perspective we want to pick out the individuals with the worst long term prognosis and focus our resources on them. In this study, we will compare the diagnostic performance of three well-established PTSD self-report instruments when predicting PTSD at 6 months after injury at 80% sensitivity, aiming to evaluate whether the two shorter instruments, the SPAN [10] and the TSQ [11], perform as well as the longer instrument, the IES-R [12].

## Method

### Ethics statement

Medical ethical approval was obtained from the local institutional review boards of the Academic Medical Centre and the VU medical centre, Amsterdam, The Netherlands.

### Participants

Participants were recruited from September 2005 to March 2009 for participation in a prospective study on the psychiatric morbidity following traumatic injury (for more details on the setting, see also Mouthaan et al. [18]). All adult patients presenting at a level-1 trauma centre in Amsterdam, The Netherlands, with injuries sustained in a traumatic event according to the DSM-IV A1-criterion for PTSD [19] and with sufficient understanding of the Dutch language were eligible for participation. Patients were excluded if they had injuries due to deliberate self-harm, an organic brain condition, current psychotic symptoms or disorder, bipolar disorder or depression with psychotic features, moderate to severe traumatic brain injury (according to the Glasgow Coma Scale score of less than 13) [20], or if they permanently resided outside the Netherlands.

### Procedure

Research assistants selected eligible patients for participation from the hospitals' registrations and contacted them within 72 hours of the injury in-hospital or by telephone. Participants provided oral and written informed consent prior to data collection and completed an extensive clinical and self-report examination of psychological symptoms, including the index tests described in this study, within 8 weeks of their injury. All patients received clinical assessments of PTSD at 6 months. These were performed at the Centre for Anxiety Disorders of the Academic Medical Centre, at bedside or at the private home of the patient by master's- and doctoral level assessors who were blind to the outcomes of the baseline tests. Formal training in the Clinician Administered PTSD Scale (CAPS) [21] was provided to all interviewers by the original translators of the Dutch version of the CAPS 5.0 of the Research Centre for Military Mental Health Care, Utrecht, The Netherlands, and by experienced clinicians from the Centre for Anxiety Disorders of the Academic Medical Centre, Amsterdam, The Netherlands.

### Measures

#### PTSD self-report instruments (index tests)

The 4-item SPAN (Startle, Physiological arousal, Anger, and Numbness) is derived from the 17-item Davidson Trauma Scale (DTS), which assesses frequency and severity of PTSD symptoms over the past week [22]. The items are rated on a 5-point scale from 0 (not at all distressing) to 4 (extremely distressing) with a total score range of 0–16. The Dutch translation of the DTS, including the SPAN items, has been previously reported to show high agreement with the Structured Interview for PTSD (SI-PTSD) [23], [24]. In our sample, the SPAN correlated moderately (Pearson *r* = .65, *p*<.001) with a concurrent CAPS assessment that was collected as a part of the larger prospective study [18]. Missing items were replaced with the mean item response (1 case with 1 missing item). Previous studies found sensitivity varying between 0.79–0.84 and specificity varying between 0.80–0.91 in relation to a concurrent PTSD diagnosis [10], [25], [26]. The only prospective study of the SPAN found a cut-off of 10 at 1 week post-trauma to be predictive of 2 month PTSD with a sensitivity and specificity of 0.86 [24].

The TSQ is a 10-item self-report scale with a yes/no-response format and assesses the presence of 5 intrusion items (e.g., “Upsetting dreams about the event”) and 5 hyperarousal items (e.g., “Difficulty falling or staying asleep”) over the past week [11]. It was adapted from the PTSD Symptom Scale-Self-Report Version (PSS-SR) [27]. The TSQ, translated into Dutch by the Research Group Psychotrauma of the Centre for Anxiety Disorders, shows adequate agreement with a concurrent CAPS assessment in the present study (Pearson *r* = .72, *p*<.001). Missing data were replaced with the scale mean (6 cases with 1 missing item). At a cut-off of 6 symptoms in any combination the TSQ showed a sensitivity/specificity of 0.86/0.93 in predicting PTSD in rail crash survivors at 6–12 months post-trauma and a sensitivity/specificity of 0.76/0.97 in crime victims within 1 month post-trauma [11]. Walters et al. [28] replicated this cut-off in a sample of emergency unit patients with a sensitivity/specificity of 0.85/0.89 for future PTSD at 1 month and of 0.88/0.78 for 6 month PTSD. At a cut-off of 7, the Dutch version of the TSQ showed a sensitivity and specificity of 0.87 and 0.69 respectively in a sample of civilian trauma survivors for future PTSD at 1 month [29].

The 22-item IES-R measures intrusion (8 items, e.g., “Any reminder brought back feelings about it”), avoidance (8 items, e.g., “I tried not to talk about it”), and hyperarousal (6 items, e.g., “I felt watchful and on guard”) [12]. The items are scored on a 5-point scale, from 0 (not at all) to 4 (extremely), with respect to how distressing each item has been in the past week. Total scores range from 0–88 with higher scores representing greater severity. In the present study, both the subscales and the total score of the Dutch IES-R, translated by the Research Group Psychotrauma of the Centre for Anxiety Disorders, show moderate to adequate similarities with the subscales and total score of the CAPS (Intrusion: *r* = .79, Avoidance: *r* = .57, Hyperarousal: *r*  = .65, total score: *r* = .75, all *p*<.001). Missing responses were replaced with the individual subscale mean (18 cases with 1 missing item, 6 cases with 2 missing items and 1 case with 3 missing items). Proposed cut-offs to indicate probable concurrent presence of PTSD were 19-20 in adolescent floods and mudslides survivors with a sensitivity/specificity of 0.86/0.84 [30], 22 in treatment-seeking substance abusers with a sensitivity/specificity of 0.92/0.57 [31] and in psychiatric patients at a sensitivity/specificity of 0.95/0.80[32], 24/25 in survivors of terrorist and natural disasters with a sensitivity of 0.83 and 1.00 and specificity of 0.67 and 0.84 [33] 33 in treatment-seeking and community-sample military veterans with a sensitivity/specificity of 0.91/0.82 [34], and an item mean score of 1.6, which corresponds to a cut-off score of 35, in acute lung injury survivors with a sensitivity/specificity of 1.00/0.85 [35].

#### Clinical interview

The 30-item semi-structured interview of the CAPS was used to obtain the final diagnosis of PTSD [21]. The CAPS is generally considered to be the best reference standard for diagnosing PTSD [36]. Frequency and intensity of the 17 DSM-IV symptoms of PTSD are scored from 0-4, with higher scores indicative of more severe symptoms. The internal consistency of the scales in the Dutch translation of the CAPS is good to excellent (Intrusion: α = 0.63, Avoidance: α = 0.78, Hyperarousal: α = 0.79, CAPS total: α = 0.89) [37]. In the current study, the CAPS subscales and total score showed similar high internal consistency (Intrusion: α = .91, Avoidance: α = .83, Hyperarousal: α = .86, CAPS total: α = .95). Missing items were replaced with the mean item response of the corresponding subscale (5 cases with 1 missing item, 1 case with 2 missing items and 1 case with 3 missing items). Inter-rater reliability was high (ICC = .98, 95% CI = .95-.99). We used the rule of Weathers et al. [38] to establish a PTSD diagnosis, in which symptoms need at least a frequency of 1 and intensity of 2 with a total score of at least 45 points.

### Analyses

Receiver operating characteristic (ROC) curves were computed for the total scores of the SPAN, the TSQ and the IES-R in relation to the 6 month PTSD diagnosis as the reference standard. As our main analysis, we performed paired *t*-tests to test for significant differences in areas under the curve (AUCs) using the method of Hanley and McNeil [39], which accounts for the paired data (subjects received all instruments under evaluation) within our study. Across all analyses, p-values of *P* = 0.05 (two-tailed) were considered to indicate statistical significance. Second, to illustrate the clinical implications of the three self-report instruments in predicting PTSD, we compared the values for specificity at 80% sensitivity. We calculated the exact specificity for 80% sensitivity by means of linear interpolation between the two data points directly before and after the 80% sensitivity. PPV, NPV and cut-off values are reported that correspond most closely to 80% sensitivity.

## Results

### Participant characteristics

**Figure 1:**
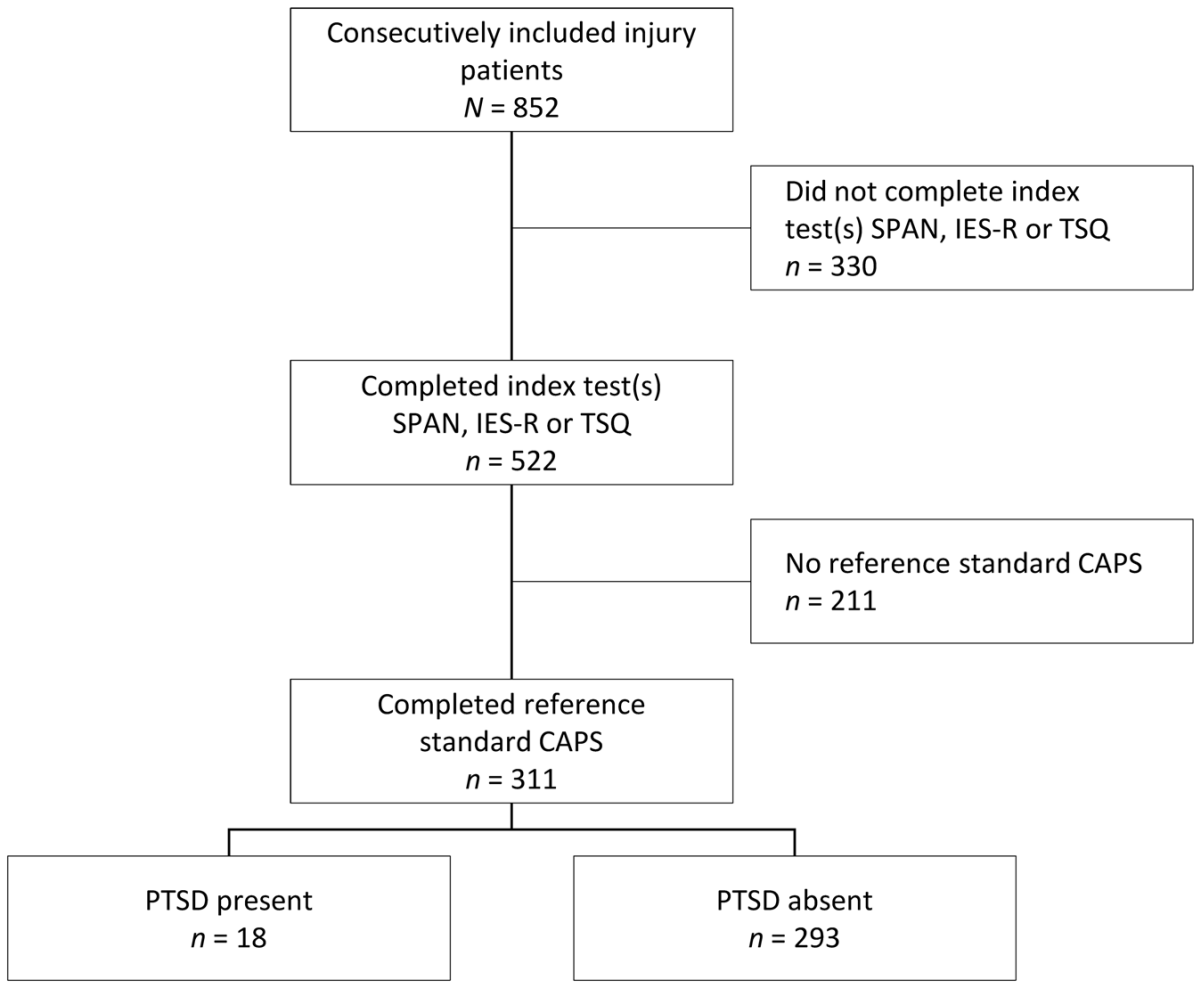
Flow chart of participants for screening for 6 month PTSD.

Of the total study sample of 852 consecutively included injury patients, 311 patients (36.5%) were included in the final sample. Patients who did not complete any of the index tests (38.7%, *n* = 330) or did not attend the 6 month assessment of PTSD (24.8%, *n* = 211) were excluded from the final sample. Figure 1 shows a flow chart of the study sample. Patients completed the SPAN, TSQ and/or the IES-R at a median of 23 days post-injury (Inter Quartile Range = 10–16) and were clinically assessed for a final diagnosis of PTSD at 6 months. Compared to the excluded patients (*n* = 541), participants were older (mean = 44.9 (s.d. = 15.9) versus mean = 42.6 (s.d. = 15.8); *t* = −2.1, d.f. = 850, *P* = .037), more often female (39.9% versus 32.9%; *χ* ^2^ = 4.2, d.f. = 1, *P* = .041), more often had a Dutch cultural background (89.3% versus 76.0%; *χ* ^2^ = 21.9, d.f. = 1, *P*<.001), and were less often diagnosed with PTSD at 6 months (5.8% versus 13.7%; *χ* ^2^ = 8.4, d.f. = 1, *P* = .004).

Eighteen patients (5.8%) of the final sample were diagnosed with 6 month PTSD. In addition, 22 patients (7.1%) were diagnosed with major depressive episode (MDE) and 24 (7.8%) with an anxiety disorder (AD) other than PTSD. Of the patients with PTSD, nine were diagnosed with comorbid MDE, two with comorbid AD and one with both. Thirty-seven patients reported having had contact with a professional mental health caregiver (i.e., psychologist, psychiatrist, social worker, victims aid worker) in the past 6 months, four of which were diagnosed with 6 month PTSD. Sixty-five patients in the final sample (20.9%) were offered a brief web-based early intervention within the first month of injury, aimed at preventing PTSD symptoms, as part of a randomized controlled trial [40]. Except for more prior traumatic experiences (*t* = −2.05, d.f. = 308, *P* = 0.011), there were no baseline differences between patients with and without PTSD. Table 1 presents baseline characteristics of participants and differences in baseline variables for patients with and without 6 month PTSD.

**Table 1:** Baseline characteristics of the total sample and participants with and without 6 month PTSD.

Variables	All participants (*n* = 311)	PTSD (*n* = 18)	Non-PTSD (*n* = 293)	*P* a
Age, mean (s.d.)	44.9 (15.9)	40.8 (10.4)	45.2 (16.1)	0.256
Male gender, No. (%)	187 (60.1)	12 (66.7)	175 (59.7)	0.559
College or university degree, No. (%)	75 (24.3)	6 (33.3)	69 (23.7)	0.624
Married or cohabitating, No. (%)	131 (42.1)	8 (44.4)	123 (42.0)	0.837
Country of origin: Netherlands, No. (%)	276 (89.3)	13 (76.5)	263 (90.1)	0.078
Prior traumatic events, mean (s.d.)	2.9 (2.2)	4.2 (1.7)	2.8 (2.2)	0.011
Traumatic event, No. %:				0.382
Road traffic accident	200 (64.3)	13 (72.2)	187 (63.8)	
Fall from height	47 (15.1)	-	47 (16.0)	
Work-related accident	36 (11.6)	3 (16.7)	33 (11.3)	
Assault/abuse	8 (2.6)	1 (5.6)	7 (2.4)	
Other (e.g., burn accident, plane crash, recreational accident)	20 (6.4)	1 (5.6)	19 (6.5)	
Hospital admission, No. (%)	204 (70.3)	10 (58.8)	194 (71.1)	0.284
Days hospitalized, mean (s.d.)	5.3 (8.4)	4.8 (8.6)	5.3 (8.4)	0.810
ICU stay, No. (%)	31 (10.5)	1 (5.9)	30 (10.8)	0.524
Injury Severity Score, mean (s.d.)	9.7 (9.8)	6.6 (7.1)	9.9 (9.9)	0.181
Glasgow Coma Score, mean (s.d.)	14.3 (2.3)	14.1 (3.2)	14.3 (2.3)	0.680

PTSD, Posttraumatic Stress Disorder; ICU, Intensive Care Unit.

a Chi-square tests were used to test for differences in categorical variables and independent samples *t* tests were used for continuous measures.

### Accuracy of the SPAN, TSQ and IES-R as screening instruments for PTSD

**Figure 2:**
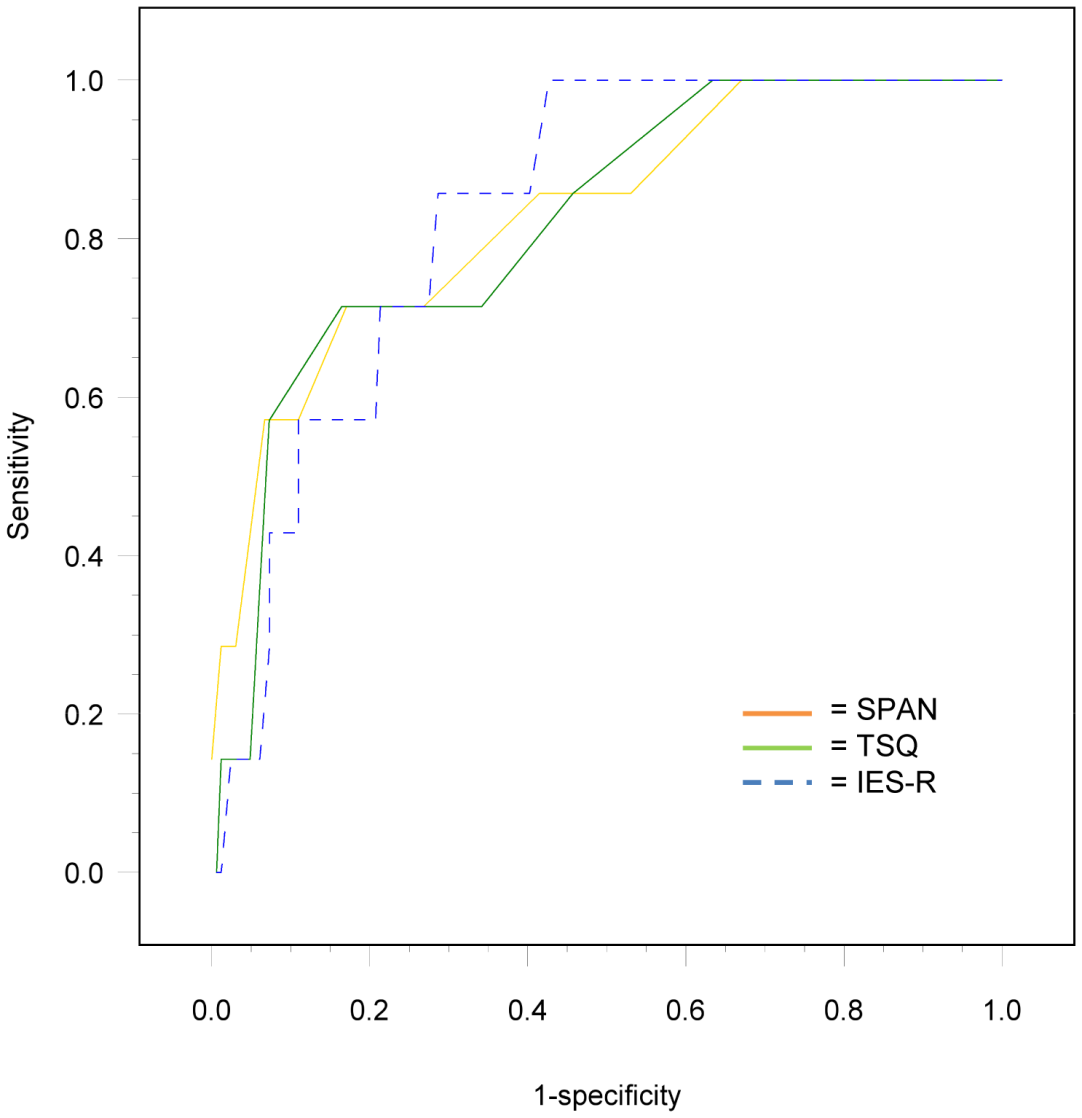
ROC curves of the SPAN, TSQ and IES-R for 6 month PTSD. Note: ROC curves represent original sensitivity and specificity values using linear interpolation between the observed data points. ROC, Receiver Operating Characteristic; SPAN, Startle, Physiological Arousal, Anger & Numbness; TSQ, Trauma Screening Questionnaire; IES-R, Impact of Event Scale-Revised; PTSD, posttraumatic stress disorder.

AUCs of the SPAN (0.83, 95% CI = 0.66–1.00), TSQ (0.82, 95% CI = 0.66–0.98) and IES-R (0.83, 95% CI = 0.72–0.94) were adequate (see Figure 2). There were no statistical significant differences between the AUCs of the SPAN, TSQ and IES-R (*P* SPAN-TSQ = 0.84, *P* SPAN-IES-R = 0.97, *P* TSQ-IES-R = 0.85). Figure 2 shows the ROC curves of the original data points of sensitivity and specificity values of the SPAN, TSQ and IES-R for 6 month PTSD using linear interpolation between the data points.

The specificity of the SPAN at 80% sensitivity was 64% (Table 2). This specificity indicates that in a population of 1,000 patients and assuming a prevalence of PTSD of 6%, the number of subjects receiving a false positive SPAN result will be 338. The cut-off most adjacent to 80% sensitivity was 4. PPV at this cut-off was 8% and NPV 99%, indicating an 8% chance of 6 month PTSD at a positive score and a 99% chance of no PTSD at a negative score. At 80% sensitivity, the TSQ's specificity was 59%, indicating that out of a 1,000 patients, 385 patients would unjustly score positive. The cut-off for the TSQ that most closely corresponded to 80% sensitivity was 5 (PPV = 0.19, NPV = 0.98). The specificity of the IES-R at 80% sensitivity was 72%, which means 263 false positives. A cut-off of 23 corresponded best with this sensitivity (PPV = 0.15, NPV = 0.99). There were no significant differences in specificities between tests.

**Table 2:** Specificity values of the SPAN, the TSQ and the IES-R at 80% sensitivity, and corresponding cut-off values, PPVs and NPVs.

		Accuracy for corresponding cut-off		
Index test	Specificity†	Cut-off	PPV	NPV
SPAN	0.64	4	0.08	0.99
TSQ	0.59	5	0.19	0.98
IES-R	0.72	23	0.15	0.99

SPAN, Startle, Physiological Arousal, Anger & Numbness; TSQ, Trauma Screening Questionnaire; IES-R, Impact of Event Scale-Revised; PPV, positive predictive value; NPV, negative predictive value.

† Values for specificity were calculated using linear interpolation at 80% sensitivity.

## Discussion

### Summary of the findings and comparison to previous studies

In this study, we compared the accuracy of three widely used early PTSD risk screening instruments in predicting a PTSD diagnosis at 6 months in injury victims. The results showed that with AUCs varying between 0.82–0.83, all instruments adequately distinguished between individuals with and without PTSD at 6 months. As we aimed for high sensitivity (80%) in order for these instruments to not miss potential clinical cases of PTSD, the specificities were modest for all instruments. This implies that while the instruments are adequate in identifying PTSD cases, they are poor in identifying non-cases. Thus, the instruments could well be used as a first selection step of possible cases, but a second, more comprehensive, diagnostic examination is needed to identify individuals in need of treatment. Importantly, the specificities did not significantly differ between tests, suggesting that the briefer, SPAN and TSQ, are as accurate as the longer, IES-R.

Our results concerning the accuracy of the SPAN, TSQ and IES-R are in line with those found in other studies that focused on screening for future PTSD [17]. Specificity results are often lower in replication studies as compared to the original validation studies, in which the items of the instruments are selected based on the performance in that particular population [29], [41]–[43]. Our proposed cut-offs of 4 for SPAN, 5 for TSQ and 23 for IES-R, best corresponding to 80% sensitivity, slightly differ from the cut-offs previously published [10], [11], [24]–[26], [28]–[35]. Our cut-off of 4 for the SPAN is much lower than found in a previous study administering the SPAN at 10 days after the trauma [24]. Perhaps the later timing of administration in our study (i.e., 3 weeks following trauma) explains this difference. At 3 weeks most initial PTSD symptoms, such as re-experiencing and hyperarousal symptoms that are usually found in the first weeks after trauma [17], are likely to have decreased naturally. Previous cut-offs reported for the TSQ were 6 [11], [28] and 7 [29]. Our cut-off of 23 for the IES-R is the first prognostic cut-off to be published, but matches the range of diagnostic cut-offs of 22 to 25 found previously [30]–[32], with the exception of studies of veterans [34] and acute lung injury victims [35] who reported cut-offs of 33 and 35 respectively.

It is important to consider that a PTSD incidence at 6% is low, which leads to low PPVs. In our sample, the highest chance of someone with a positive result developing PTSD at 6 months was 19%. It is an issue found in several previous screening studies, especially within injury populations [24], [28], [32], [44]. As our PPVs imply, at most only one in five persons scoring positive on the instruments will actually develop PTSD. This hampers the usefulness of screening initiatives in these types of population. Among patients who did not complete the early PTSD risk screening instruments (*n* = 330), PTSD prevalence was double that of the final sample (13.7%). This could indicate a reluctance to screening or research participation among patients with a higher risk of later PTSD. This issue has also been raised previously [41] and limits the generalizability of the findings to samples with a higher PTSD prevalence.

Previous studies have shown good results in identifying individuals at risk for depressive and anxiety disorders using screeners of 5, 3, 2 or even a single item [45]–[47]. Our finding that the short instruments perform equal to the longer one are in line with these results. However, comparable to our results, these screeners generally suffered from moderate to low specificity (44% to 77%) [45]–[47]. Thus, it may be worthwhile to evaluate the diagnostic accuracy of even shorter early PTSD risk screening instruments than in the current study, but only when followed by a comprehensive clinical evaluation, to rule out any false positives as a result of the lower specificity.

### Strengths and limitations

A strength of our study is that we tested the accuracy of three instruments that are already in use globally in various populations and practices. Our study employed a comparative accuracy design, meaning that all patients received all tests under evaluation as well as the best available reference standard. Such a design has both higher validity and greater efficiency than comparing instruments between samples. Higher validity comes from the fact that all external factors can be kept constant when comparing the instruments; efficiency is enhanced because of the within patient comparisons that can be made similar to a paired t-test. This knowledge helps us make informed decisions on which instrument to best choose in a certain situation or population. Because these instruments do not rely on information specific to a particular trauma population (e.g., injury characteristics or the context in which the trauma occurred), but instead on symptoms, they may be useful for multiple groups or situations. A strong point of our sample is that, compared to other studies of screening in acutely traumatised populations [24], [28], [44], ours included a heterogeneous, consecutively included sample of injury victims, reflecting the broad range of injury victims of a level-1 trauma centre. We included various accident and assault victims, admitted to the hospital for a long period or immediately released. Finally, this study was the first to provide a prognostic cut-off for the IES-R and the first to examine the Dutch versions of the TSQ and the SPAN in a prospective design and using a consecutively included sample.

A limitation is that the screening instruments were administered quite late, at around 3 weeks following injury. This limits the generalizability of our findings to screening efforts in the immediate aftermath of traumatic events. Additionally, we were unable to collect a clinical PTSD diagnosis of 211 patients at 6 months, whose results on the index tests could not be included. However, post-hoc analyses revealed no significant differences in demographics, trauma history or scores on the screening instruments between participants and these dropouts. The determination of specificity values was hampered due to the relatively small sample size in the study. The total number of patients with a final diagnosis of PTSD was relatively low in this study. This hampered in particular the exact determination of cut-off values to obtain a specific value of sensitivity. Due to the low PTSD incidence in our sample, the ROC curves were surrounded with considerable uncertainty, which may have led to small, but potentially significant, differences between instruments remaining undetected.

### Clinical implications

An important clinical implication of our study is that shorter instruments perform as well as longer ones in early PTSD risk screening. The use of shorter instruments may enhance the response rate to these screening instruments [48]. This means more individuals could be monitored or even referred for follow-up. Our results also imply that early screening cannot be used in clinical practice without adequate diagnostic follow-up. As a triage strategy, early screening can assist in filtering out anyone in need of PTSD treatment, without having to conduct time-consuming clinical interviews in low-risk individuals. This may not be problematic, since mental health treatment is usually preceded by a more thorough clinical interview.

### Research implications

Future studies should investigate the potential disadvantages of early screening, such as the hazard of stigmatizing individuals when wrongly identifying them as potential PTSD cases. Lastly, the design and method of our study of comparing the diagnostic accuracy within the same patients and at a fixed sensitivity level should also be replicated in trauma populations with a higher PTSD prevalence, such as victims of rape or violence [49], to examine whether the instruments perform equally accurate in samples with a higher proportion of PTSD cases.
